# Gender Differences Time Trends for Metabolic Syndrome and Its Components among Tehranian Children and Adolescents

**DOI:** 10.1155/2012/804643

**Published:** 2012-04-19

**Authors:** Maryam Barzin, Farhad Hosseinpanah, Hamidreza Saber, Parvin Sarbakhsh, Kobra Nakhoda, Fereidoun Azizi

**Affiliations:** ^1^Obesity Research Center, Research Institute for Endocrine Science, Shahid Beheshti University of Medical Sciences, 1985717413 Tehran, Iran; ^2^Endocrine Research Center, Research Institute for Endocrine Science, Shahid Beheshti University of Medical Sciences, 1985717413 Tehran, Iran

## Abstract

*Aims*. To investigate the trend of metabolic syndrome and its components in Tehran children and adolescents during a median followup of 6.6 years. *Methods*. Data from 1999–2001 (phase I), 2002–2005 (phase II), and 2006–2008 (phase III) of the Tehran, Lipid and Glucose Study were analyzed (*n* = 5439; age 6–18 years) for the trend of metabolic syndrome (MetS) and its components. General estimation equation (GEE) models were used to analyze this correlated data. *Results*. The crude prevalence of MetS for boys at baseline was 13.2%, which increased to 16.4% in the third phase. In girls, the prevalence of Mets decreased from 11.8% at baseline to 6% during followup. The odd ratios (OR) of obesity over the whole study period were raised in both sexes. The OR of abdominal obesity increased significantly in boys, but no change was observed in girls. No significant OR was observed in boys, while OR for MetS was shown to have a decreasing trend in girls during the followup. In the three time points, the ORs of MetS decreased significantly in girls but no significant difference was observed in boys. *Conclusion*. Inspite of increasing trend for obesity in both sexes, the trend of MetS decreased in girls and was relatively stable in boys, in Tehranian children, and adolescents.

## 1. Introduction

The metabolic syndrome (MetS) is defined as a clustering of metabolic risk factors including central obesity, hyperglycemia, dyslipidemia, and hypertension [1]. In recent decades, obesity and metabolic risk factors among children and adolescents have been much focused on several studies showing that increasing obesity and MetS in this population is associated with a number of adverse consequences in adulthood including type 2 diabetes mellitus and coronary heart disease, most likely due to overproduction of inflammatory mediators and insulin resistance [2–4].

While current estimates indicate a 2% to 9% prevalence for MetS in US adolescents [5], 14.1% of Iranian children have MetS based on ATP III criteria resulting from the increasing prevalence of overweight and abdominal obesity among Iranian children and adolescents [6, 7]. Previous studies also demonstrated higher triglyceride and lower HDL-C levels in Iranian adolescents compared to their American counterparts [8, 9]. This increased prevalence would necessitate the need for identifying the time trend of Mets and its components in our population. Although the prevalence and associated risk factors of MetS have been widely studied in recent decades [10–12], much less is known in regard to changes in risk factor status over a longer period of time during childhood and adolescence, particularly in developing countries like Iran.

A few studies have evaluated the tracking of MetS and its components altogether [13–17] and most of them have provided data in support of a stability of MetS and its components in their populations [13, 14, 16]. Other studies have demonstrated an increasing trend in MetS [17] and its individual components [18–21]. In a recent study which used three independent sets of cross-sectional data with limited sample size in Tehranian adolescents, aged 10–19 years, reported an increasing trend for obesity, abdominal obesity [22]. Given the above-mentioned limitations in this study, we aimed to examine the trend of MetS and its components from childhood to adolescence, due a mean of 6.6 years of followup for the first time in Middle East region using general estimation equation (GEE) analysis. 

## 2. Materials and Methods

Subjects in this study were selected from among participants of the Tehran Lipid and Glucose Study (TLGS), a prospective study conducted to determine the risk factors and outcomes for noncommunicable diseases [23]. To summarize, 15,005 people, aged 3 years and over, residents of district-13 of Tehran underwent a baseline examination between February 1999 and August 2001. After this cross-sectional (phase 1), subjects were categorized into the cohort and intervention groups, the latter to be educated for implementation of life-style modifications. We used metabolic and anthropometric data from phases I (1999–2001), II (2002–2005), and III (2006–2008) of the TLGS. For the current study, 5439 participants, 2643 boys and 2797 girls, aged 6–18 years who had participated at least in one of three phases, were enrolled. The study was approved by the institutional ethics committee of the Research Institute for Endocrine Sciences, affiliated to Shahid Beheshti University of Medical Sciences and was conducted in accordance with the principles of the Declaration of Helsinki.

Details of the TLGS protocol and all laboratory procedures have been published elsewhere [10]. Briefly, trained interviewers collected information, using a pretested questionnaire which included demographic data and anthropometric indices. Weight, height, and waist circumference (WC) were measured using standard protocols. Body mass index (BMI) was calculated as weight in kilograms, divided by height in meters squared. A qualified physician measured blood pressure twice with the subject in a seated position after one initial measurement for determining peak inflation level using a standard mercury sphygmomanometer; the mean of two measurements was considered to be the participant's blood pressure. Fasting blood samples for the measurement of glucose and lipid concentrations were drawn after the subjects had fasted overnight. Fasting blood glucose (FBG) was measured on the day of blood collection by the enzymatic colorimetric method using glucose oxidase. Triglyceride (TG) concentrations were measured by commercially available enzymatic reagents (Pars Azmoon, Tehran, Iran) adapted to a Selectra autoanalyzer. High-density lipoprotein cholesterol (HDL-C) was measured after precipitation of the apolipoprotein B-containing lipoproteins with phosphotungstic acid.

### 2.1. Definitions

We used the definition based on Cook et al. work for definition of the MetS in children and adolescents [24]. This definition is based on criteria analogous to that of the National Cholesterol Education Program Expert Panel on Detection, Evaluation and Treatment of High Blood Cholesterol in Adult Treatment Panel III [1]; it defines MetS as three or more of the following: fasting TG ≥ 110 mg/dL; HDL cholesterol <40 mg/dL; WC ≥ 90th percentile for age and sex, according to national reference curves [25]; systolic blood pressure (SBP) and/or diastolic blood pressure (DBP) ≥90th percentile for sex, age and height, from national reference cut-off points [26]; FBG ≥ 100 mg/dL. For the subjects who were aged over 18 years after the followup, we used the criteria for MetS in adults, specified by the Joint Interim Statement (JIS) [27]. Obesity was defined based on the standardized percentile curves of BMI suggested for Iranian children and adolescents as ≥95th percentile of BMI for age and sex [6].

### 2.2. Statistic Analysis

All continuous data are expressed as mean ± SD, and categorical variables are expressed as percentages. Independent *t*-test was used to compare difference between two sexes within in each phase. The general estimation equation (GEE) use the generalized linear model to estimate more efficient and unbiased analysis of demographic variables, anthropometric indices, and biochemical variables collected in longitudinal, nested, or repeated measures designs [28, 29]. This method relies on independence across subjects to consistently estimate the variance of the proposed estimators even when the assumed working correlation structure is incorrect. Logistic regression analysis was performed using GEE method for binominal variables. Variables were adjusted for age (years), time (phase) and intervention.

All analyses were performed using SPSS for Windows (version 16; SPSS Inc., Chicago, IL, USA), and significance was set at *P* < 0.05.

## 3. Results

For the current study, we had 3,854, 3,057, and 3,441 observations in phases I, II, and III, respectively. The GEE analysis was performed with 5,439 subjects (2643 boys), aged 6–18 years, who had at least one observation in whole period the study. At baseline, girls had higher mean values for WC (*P* = 0.04), TG (*P* = 0.01), LDL, and cholesterol (*P* < 0.001) but lower SBP and FBS than boys (*P* < 0.001). At the end of followup, all factors were higher in boys, while cholesterol and HDL values were higher in girls (*P* = 0.001, Table 1).

**Figure 1:**
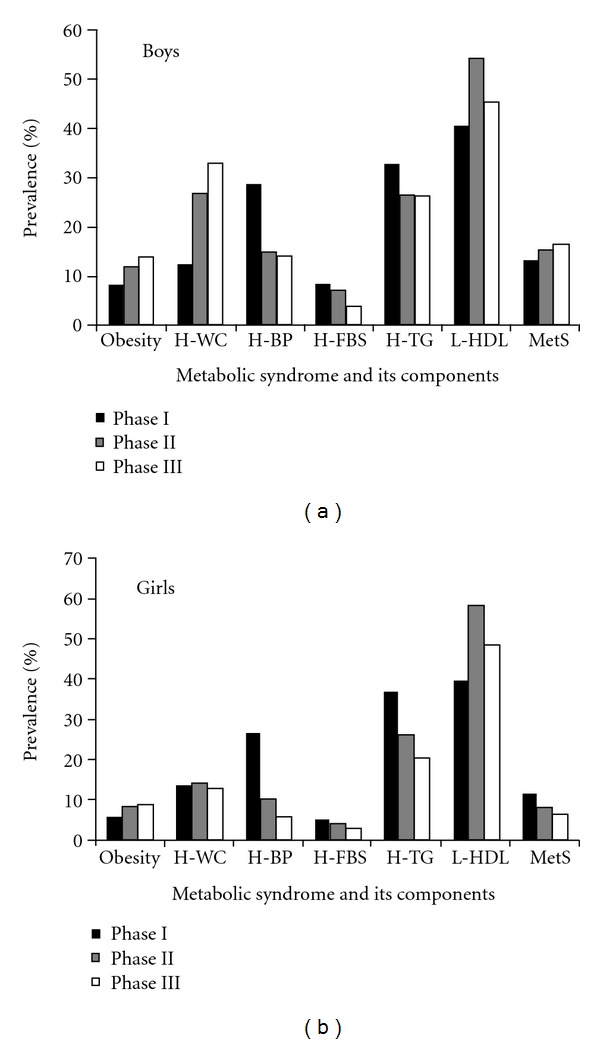
Prevalence of metabolic syndrome (MetS) and its components in boys (*n* = 2643) and girls (*n* = 2797). Obesity defined as ≥95th percentile of BMI for age and sex; high waist circumference (H-WC) ≥90th percentile for age and sex, according to national reference curves; high blood pressure (H-BP), SBP and/or DBP ≥90th percentile for sex, age and height, from national reference cut-off points; high fasting blood glucose (H-FBG), fasting glucose ≥100 mg/dL; high triglycerides (H-TG), fasting TG ≥ 110 mg/dL; low HDL cholesterol (L-HDL), HDL < 40 mg/dL.

The crude prevalence of obesity increased in both sexes during a median follow-up of 6.6 years. Abdominal obesity increased from 12.3% to 33.1 in phase III in boys, but remained fairly stable in girls. The most frequent component of MetS was low HDL at baseline (39%) that increased up to 47% for boys and 43% for girls in the third phase. High TG was the next most prevalent component that decreased from 35% and 31% at baseline to 21% and 25% in girls and boys, respectively. The prevalence of MetS was 13.2% for boys at baseline that increased to 16.4% in the third phase. In girls, the prevalence of Mets decreased from 11.8% at baseline to 6% during followup (Figure 1).

The odds of obesity over the whole study period were raised in both sexes (*P* < 0.01). While the odds of abdominal obesity increased significantly in boys (*P* < 0.001), whereas no significant change was observed in girls (*P* = NS). The odds for all other MetS component values declined towards the end of followup, except for HDL in girls. At three time points, the odds of MetS decreased significantly in girls (OR: 0.69 and 0.55 *P* < 0.001 in phases II and III, resp.) but no significant difference was observed in boys (OR: 1.1 and 0.94 *P* = NS in phases II and III resp., Table 2).

## 4. Discussion

Using GEE analysis, the results of present study conducted on 5,439 Tehranian children and adolescents, aged 6–18 years, suggest that during a median follow-up of 6.6 years, the trend of obesity increased in both sexes and for abdominal obesity increased almost three folds in boys. In spite of the increasing trend for the above factors, the trend for MetS decreased in girls and remained relatively stable in boys.

The high prevalence of obesity and MetS in childhood and adolescence has been shown to increase the prevalence of cardiovascular events in adults [30]. Childhood and adolescent MetS have evolved into a worldwide epidemic, up to 14% in Australian [31] and 11% in Italian children [32]. Findings from previous studies in Iran have revealed a higher prevalence of MetS among Iranian children (9.8% [7]) compared with western (4.2% in US [24]) and Asian countries (1.4% in Japan [33] and 1.8% in Korea [15] 6.6% in China [34]). Several previous epidemiological studies demonstrated different trends for MetS and cardiometabolic risk factors among children and adolescents [13–17], which can be explained by variety in MetS definitions, length of followup, and also specific cultural and ethnical composition of studied population, lifestyle, and public health policies. In the Young Finns Study, a follow-up study of 1769 girls and 1688 boys, aged 3–18 years old, the prevalence of MetS increased with age in boys, and in 9–12 years old age girls [17]. The results from the Quebec family study indicated that indicators of MetS are moderately stable from childhood to adolescence [16]. Based on the Fels longitudinal study, authors concluded that 8 cardiometabolic risk factors associated with the MetS were relatively stable among children and adolescents [13]. On the other hand, results from the Korean NHANES Survey on 4164 subjects, aged 10–19 years, demonstrated a decline in prevalence of MetS, despite an increase in obesity in Korean adolescents [15]. In agreement with the Korean study, despite increasing trend of obesity, we found a decreasing trend of MetS in girls, but it relatively stable in boys mostly as a result of improvement in other MetS components. Similarly, an increasing prevalence of childhood obesity has been reported around the world [35–37]. Rapidly changing dietary practices and a sedentary lifestyle have led to increasing prevalence of childhood obesity (5–19 years) in developing countries recently [38] such as Iran [22]. Moreover, recent systematic reviews and meta-analyses of available randomized trials reported nonsignificant effects of pharmacologic and behavioral treatments for reduction of overweight and obesity in children and adolescents [39, 40]. Regarding above mentioned studies, cardiometabolic risk factors were stable across time besides increasing trend of obesity in children and adolescents.

 The decreasing prevalence of cardiometabolic risk factors can be explained by the positive effects of public health interventions in terms of lifestyle behaviors and increased physical activity on unfavorable risk factors. Accordingly, a systematic review has shown that higher physical activity levels were consistently associated with an improved metabolic profile and a reduced risk for MetS and/or insulin resistance in pediatric populations [41]. We speculate that recent changes in physical activity in addition to national health care initiatives in Tehran may have had a positive impact on the prevalence of MetS. We also found some notable differences between genders in the trend of MetS, for which however we do not have an adequate explanation; it could possibly be explained by more focus of public educational programs on girls compared to boys, leading to decreasing trend of MetS in girls.

The strength of our study was the considerable sample size of children and adolescents with a long followup period over which we measured MetS and its components enabling us to assess the time effect of these risk factors in our population using the GEE model for the first time.

However, our study had several limitations. First, our subjects were from a homogeneous population, potentially limiting the generalizability of our results. Second, we did not have data regarding puberty status which have helped us to assess the effects of puberty on MetS, and third, we did not take into account some possible confounders such as physical activity, dietary habits, and socioeconomic status in our analysis.

## 5. Conclusion

In conclusion, inspite of the increasing trend for obesity in both sexes, the trend for MetS decreased in girls and remained relatively stable in boys in our population. Given the proven association between childhood and adolescence, obesity, and accuracy of cardiovascular events in adulthood, we are not sure that our observations, indicating persistency of excess weight along with reduction of cardiometabolic risk factors can be translated to decrease cardiovascular event rates in future adulthood. Further prospective studies with long-term followup are needed to answer this question.

## Figures and Tables

**Table 1:** Anthropometric and metabolic characteristics of cohort participants by sex in 3 phases.

	Phase I		Phase II		Phase III	
	Boys *N* = 1858	Girls *N* = 1995	Boys *N* = 1415	Girls *N* = 1641	Boys *N* = 1592	Girls *N* = 1849
Age (year)	12.5 ± 3.5	12.6 ± 3.6	14.2 ± 4.53	14.7 ± 4.5	16.0 ± 5.6	16.6 ± 5.4
Weight (kg)	44.7 ± 19.2	42.7 ± 15.56*	53.1 ± 21.8	48.2 ± 16.0*	59.3 ± 23.6	51.4 ± 15.7*
BMI (kg/m²)	18.9 ± 4.4	19.0 ± 4.5	20.6 ± 5.0	20.6 ± 4.5	22.0 ± 6.3	21.4 ± 4.6*
Overweight (%)	19.7	17.5	28.3	23.7*	34.3	27.2*
Obesity (%)	8.3	6*	11.9	8.4*	13.8	8.9*
WC (cm)	65.0 ± 12.5	65.7 ± 10.8*	73.6 ± 14.6	69.0 ± 11.3*	77.8 ± 15.8	69.2 ± 11.0*
Abdominal obesity (%)	12.3	13.7	26.9	14.3*	33.1	13*
SBP (mmHg)	106.2 ± 12.2	103.6 ± 11.3*	104.0 ± 13.0	98.8 ± 11.3*	104.6 ± 13.2	98.3 ± 11.3*
DBP (mmHg)	71.1 ± 9.5	71.2 ± 9.4	67.2 ± 10.0	67.2 ± 9.4	67.5 ± 10.5	64.7 ± 9.6*
FBG (mg/dL)	88.9 ± 11.3	86.9 ± 8.3*	88.7 ± 11.6	86.2 ± 7.4*	86.9 ± 7.5	84.7 ± 7.0*
TG (mg/dL)	103.7 ± 60. 7	107.9 ± 54.9*	101.6 ± 54.4	99.0 ± 47.7	105.2 ± 61.4	94.6 ± 45.9*
HDL-C (mg/dL)	44.0 ± 10.8	44.0 ± 10.5	40.8 ± 10.3	41.5 ± 10.1	42.8 ± 10.3	45.6 ± 10.4*

Data are presented as mean (SD) or percent.

BMI, body mass index; WC, waist circumference; SBP, systolic blood pressure; DBP, diastolic blood pressure; FBG, fasting blood glucose; TG, triglycerides HDL-C, high density lipoprotein cholesterol. Overweight defined as ≥85th to <95th percentile of BMI for age and sex; obesity defined as ≥95th percentile of BMI for age and sex; abdominal obesity defined as WC ≥ 90th percentile for age and sex.

*P < 0.001 (between boys and girls in each phase).

**Table 2:** Odd ratios^a^ of the incident MetS parameters in phase I, II and III.

Characteristics	phase I	phase II	phase III
Boys			
obesity	1	1.40 (1.16–1.68)*	1.49 (1.19–1.87)*
Abdominal obesity	1	2.28 (1.95–2.67)*	2.61 (2.19–3.12)*
High FBG	1	0.90 (0.69–1.18)	0.47 (0.34–0.64)*
Low HDL-C	1	1.51 (1.32–1.73)*	0.84 (0.73–0.96)*
High TG	1	0.68 (0.58–0.78)*	0.63 (0.54–0.74)*
High BP	1	0.45 (0.37–0.53)*	0.42 (0.34–0.52)*
MetS	1	1.1 (0.90–1.33)	0.94 (0.73–1.22)

Girls			
obesity	1	1.42 (1.14–1.77)*	1.41 (1.07–1.84)*
Abdominal obesity	1	1.08 (0.912–1.28)	0.98 (0.79–1.21)
High FBG	1	0.78 (0.56–1.08)	0.59 (0.42–0.83)*
Low HDL-C	1	1.89 (1.66–2.14)*	1.07 (0.93–1.22)
High TG	1	0.65 (0.67–0.75)*	0.50 (0.43–0.58)*
High BP	1	0.50 (0.43–0.58)*	0.21 (0.16–0.27)*
MetS	1	0.21 (0.16–0.27)*	0.55 (0.41–0.75)*

Obesity, BMI ≥ 95th percentile for age and sex; abdominal obesity, WC ≥ 90th percentile for age and sex; high FBG, FBS ≥ 100 (mg/dL); Low HDL-C, HDL-C < 40 (mg/dL); high TG, TG ≥ 110 (mg/dL); High BP, SBP, and/or DBP ≥90th percentile for sex, age, and height.

^a^(95% Confidence interval).

*P value <0.05 compare to phase I.
